# Supplementary material for: Common schizophrenia risk variants are enriched in open chromatin regions of human glutamatergic neurons
Source: Nat Commun. 2020 Nov 4;11:5581. doi: 10.1038/s41467-020-19319-2 (PMC7643171; doi:10.1038/s41467-020-19319-2)
Supplement: Supplementary file 1 — Supplementary Information [file 41467_2020_19319_MOESM1_ESM.pdf]

Supplementary Information

Common schizophrenia risk variants are enriched in open chromatin regions of human glutamatergic neurons

Hauberg et al.

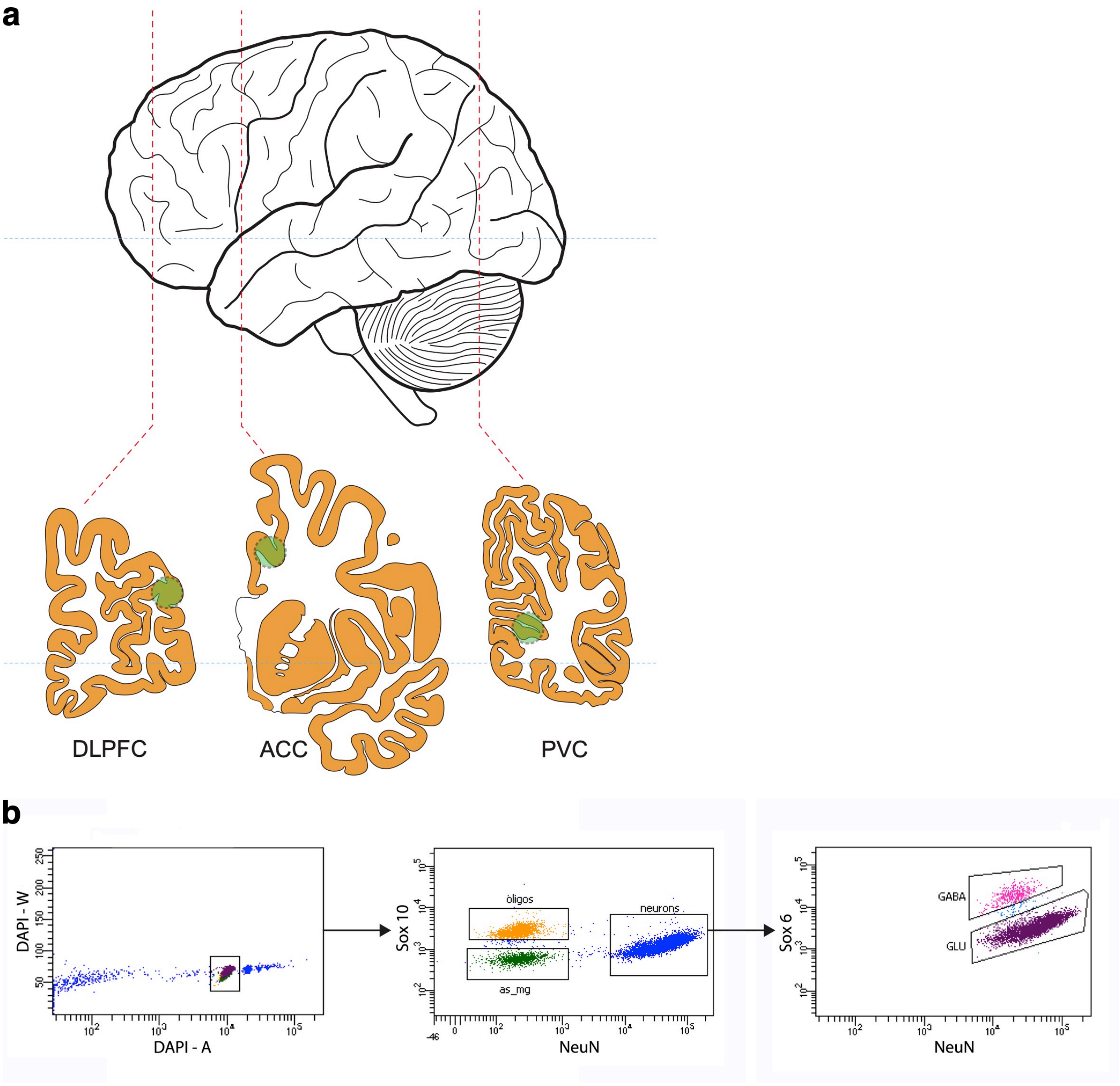

Supplementary Figure 1. Brain regions investigated in the present study and nuclei sorting. (a) The anatomical localization of the three brain regions investigated in this study. (b) Gating strategy to isolate nuclei of specific cell-type origin. DAPI+ singlets were gated and assessed for binding of both NeuN and Sox10. This allowed for the identification and subsequent collection of oligodendrocyte (DAPI+ NeuN- SOX10+) and microglia/astrocyte (DAPI+ NeuN- SOX10-) nuclei. In parallel, the NeuN+ population was gated and assessed for binding of Sox6, allowing for isolation of nuclei from both GABAergic (DAPI+ NeuN+ SOX6+) and Glutamatergic neurons (DAPI+ NeuN+ SOX6-). DLPFC: dorsolateral prefrontal cortex; ACC: anterior cingulate cortex; and PVC: primary visual cortex.

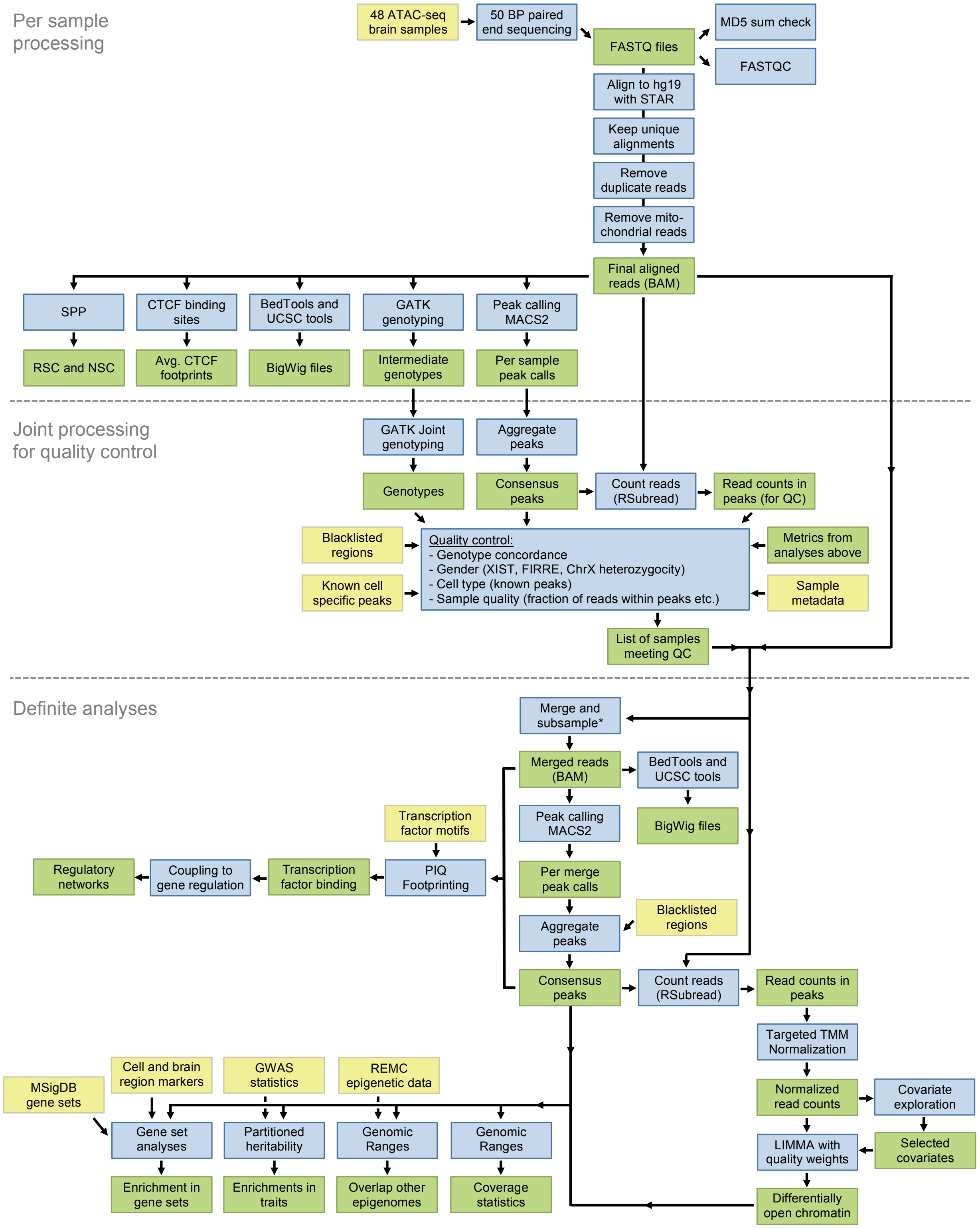

Supplementary Figure 2. Flowchart of the overall data processing. The analytical steps generally speaking fall under three different phases: 1) the per sample processing, 2) joint processing for quality control, and 3) analyses of samples meeting the quality control. Yellow: input data. Blue: analyses. Green: processed data.

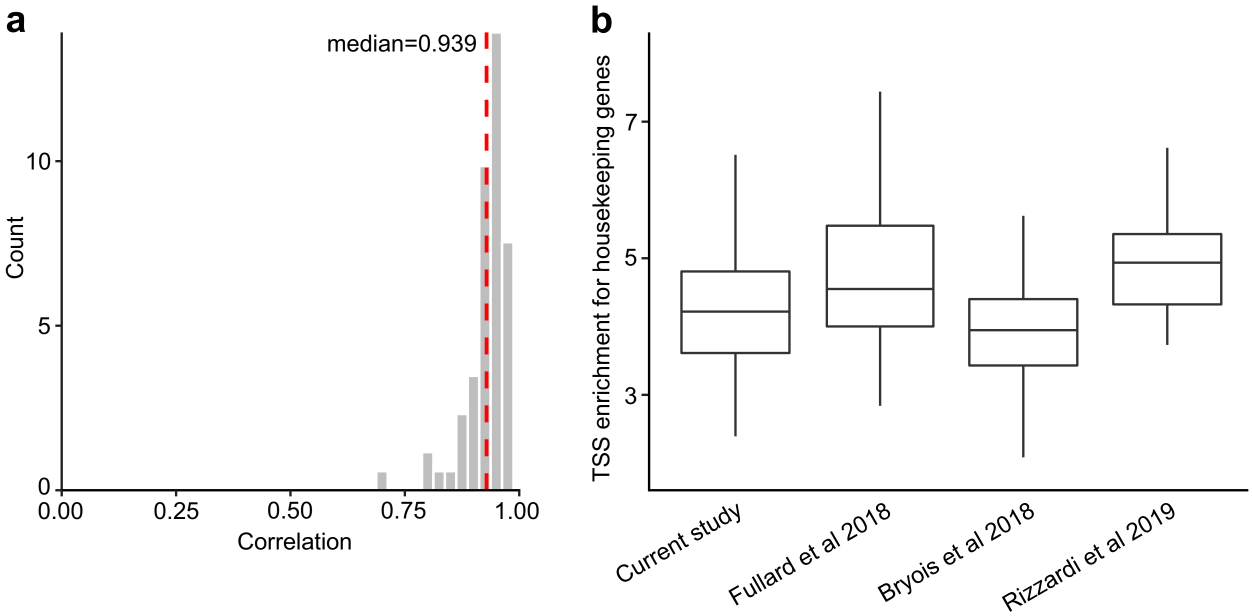

Supplementary Figure 3. Intersample correlation and TSS enrichment. (a) Correlations of raw reads counts over consecutive bins of 10,000 bp genomic regions between samples originating from the same cell type and brain region. (b) Comparison of TSS enrichment in housekeeping genes for our dataset and three additional postmortem human brain ATAC-seq datasets. The center shows the median, the box shows the interquartile range, whiskers indicate the highest/lowest values within 1.5x the interquartile range, and potential outliers from this are shown as dots. From left to right, the boxes illustrate 47, 115, 247, and 22 individual samples. Note that the double sorting employed in the current study puts more stress on the nuclei than a single sorting step (Fullard and Rizzardi) or simply using bulk tissue (Bryois).

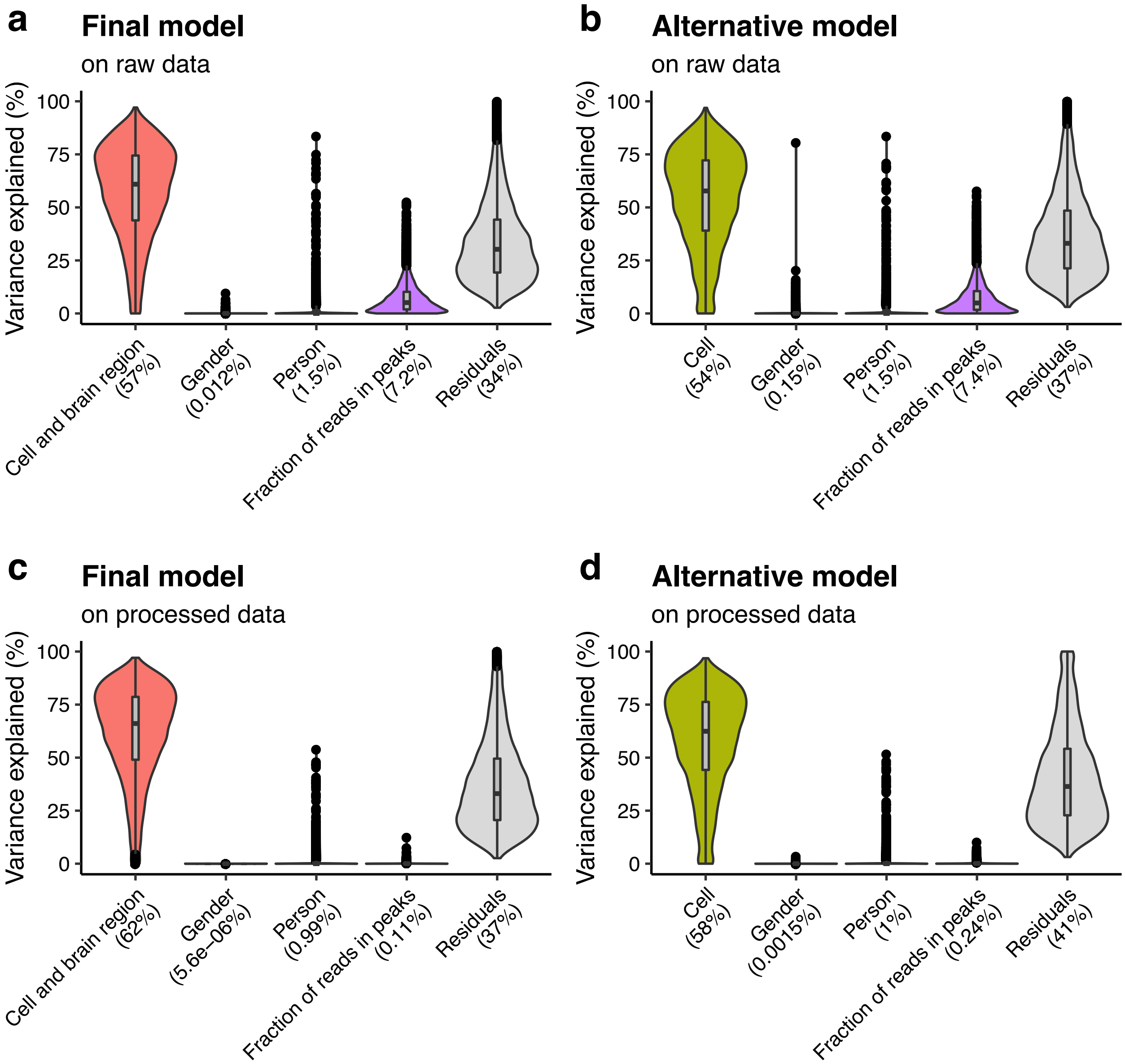

Supplementary Figure 4. Variance partition analysis of chromatin accessibility. (a) Proportion of variation in chromatin accessibility based on the statistical model presented in the main text. Here cell type and brain region jointly account for 57% of the variance. **(b)** An alternative model without considering brain region. Cell type alone accounted for 54% of the variance, signifying the comparatively little contribution of brain region to chromatin accessibility compared to cell type. **(c,d)** Proportion of variation in chromatin accessibility after residualization of gender and fraction of reads in peaks. This illustrates that the residualization mostly removes the variance from these confounders. Fraction of reads in peaks can be considered a signal to noise parameter. For computational feasibility, these figures were based on a random subset of 10,000 OCRs.

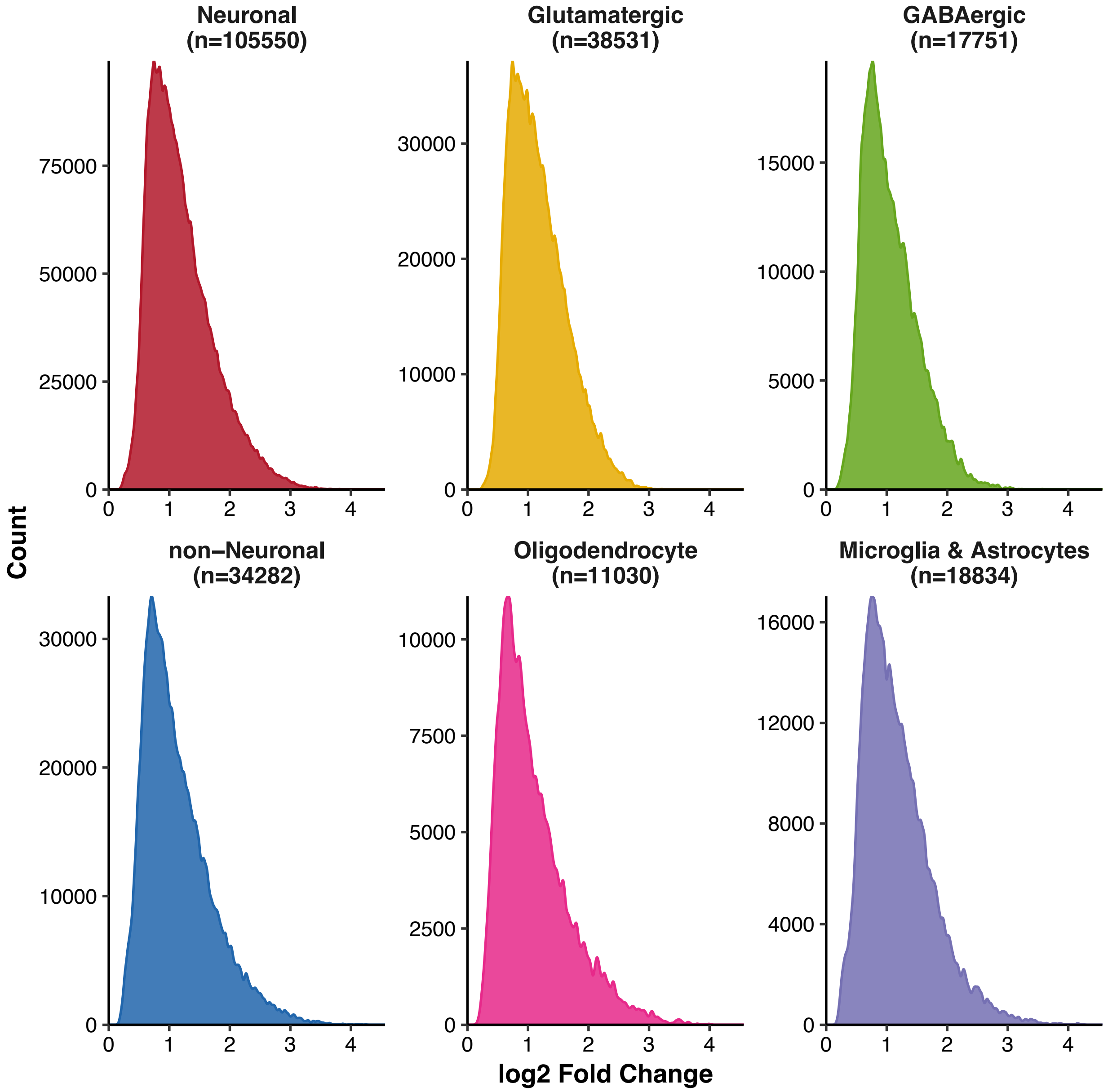

Supplementary Figure 5. Fold change metrics for cell-specific open chromatin regions. The distribution of fold change estimates of chromatin accessibility for cell-specific OCRs. These OCRs are chosen as an intersection OCRs significant in multiple comparisons (**Online Methods**), and we report the fold change estimate for the comparison with the least significant *P*-value.

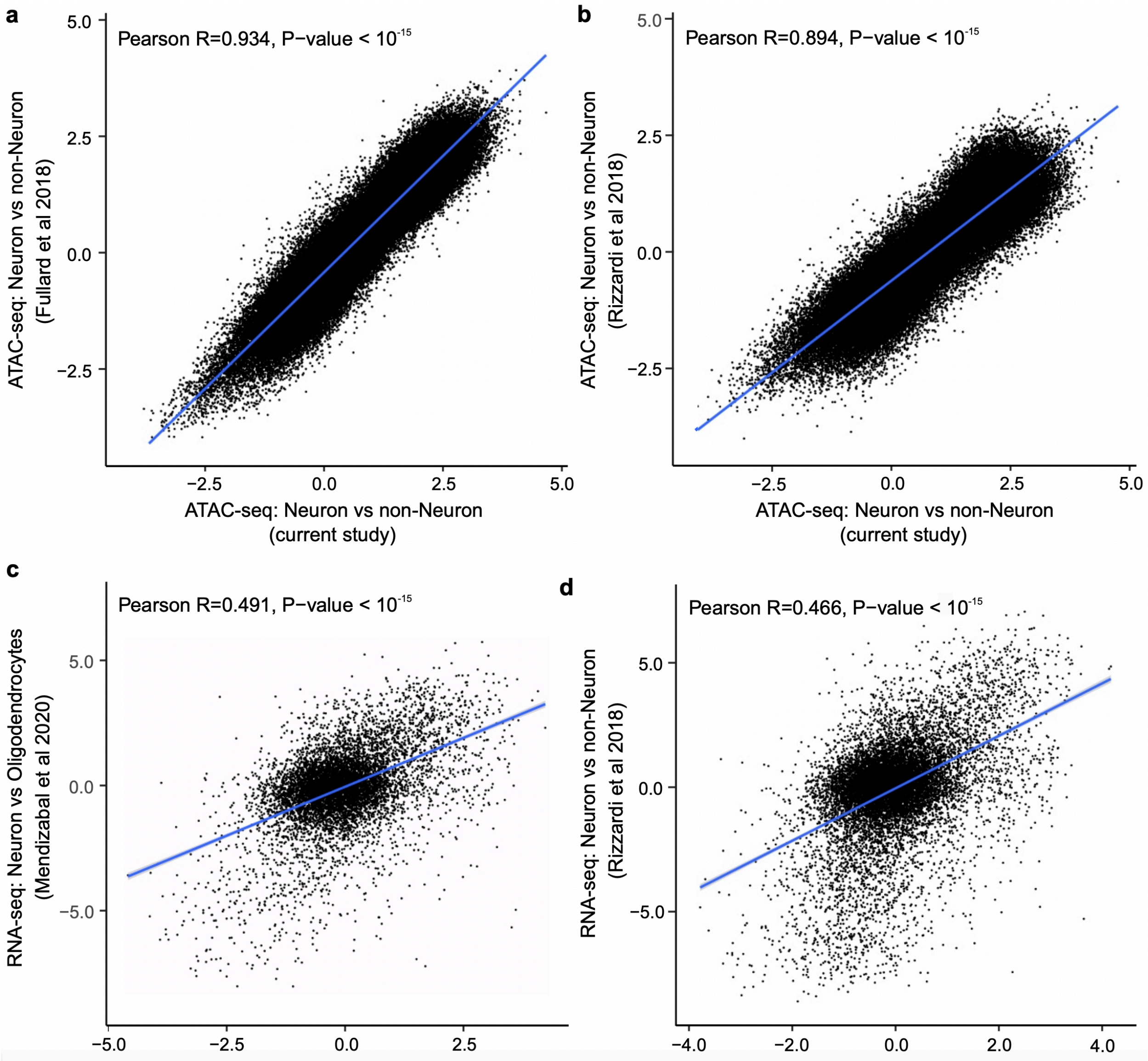

Supplementary Figure 6. Comparison of cell specificity across RNA/ATAC-seq studies. The cell specificity of OCRs and gene expression was assessed using fold change concordance (log2 values) between our ATAC-seq dataset (fold changes for comparison between neuronal vs non-Neuronal / Oligodendrocyte samples) and external ATAC-seq / RNA-seq datasets. For external ATAC-seq datasets, correlation was calculated on overlapping OCRs (minimum 25% overlap). For external RNA-seq datasets, correlation was calculated between fold change of chromatin accessibility of promoter OCRs from our ATAC-seq and fold change of gene expression of corresponding genes from external RNA-seq study. This simplistic approach to correlating ATAC-seq to RNA-seq is not expected to yield high correlations, as distal elements are ignored and as OCRs include both enhancers and silencers. (a) ATAC-seq of NeuN+/- samples from 14 brain regions. (b) ATAC-seq from NeuN+/- samples from dorsolateral prefrontal cortex and nucleus accumbens. (c) RNA-seq from NeuN+/Olig2+ samples from dorsolateral prefrontal cortex. (d) RNA-seq from NeuN+/- samples from dorsolateral prefrontal cortex and nucleus accumbens.

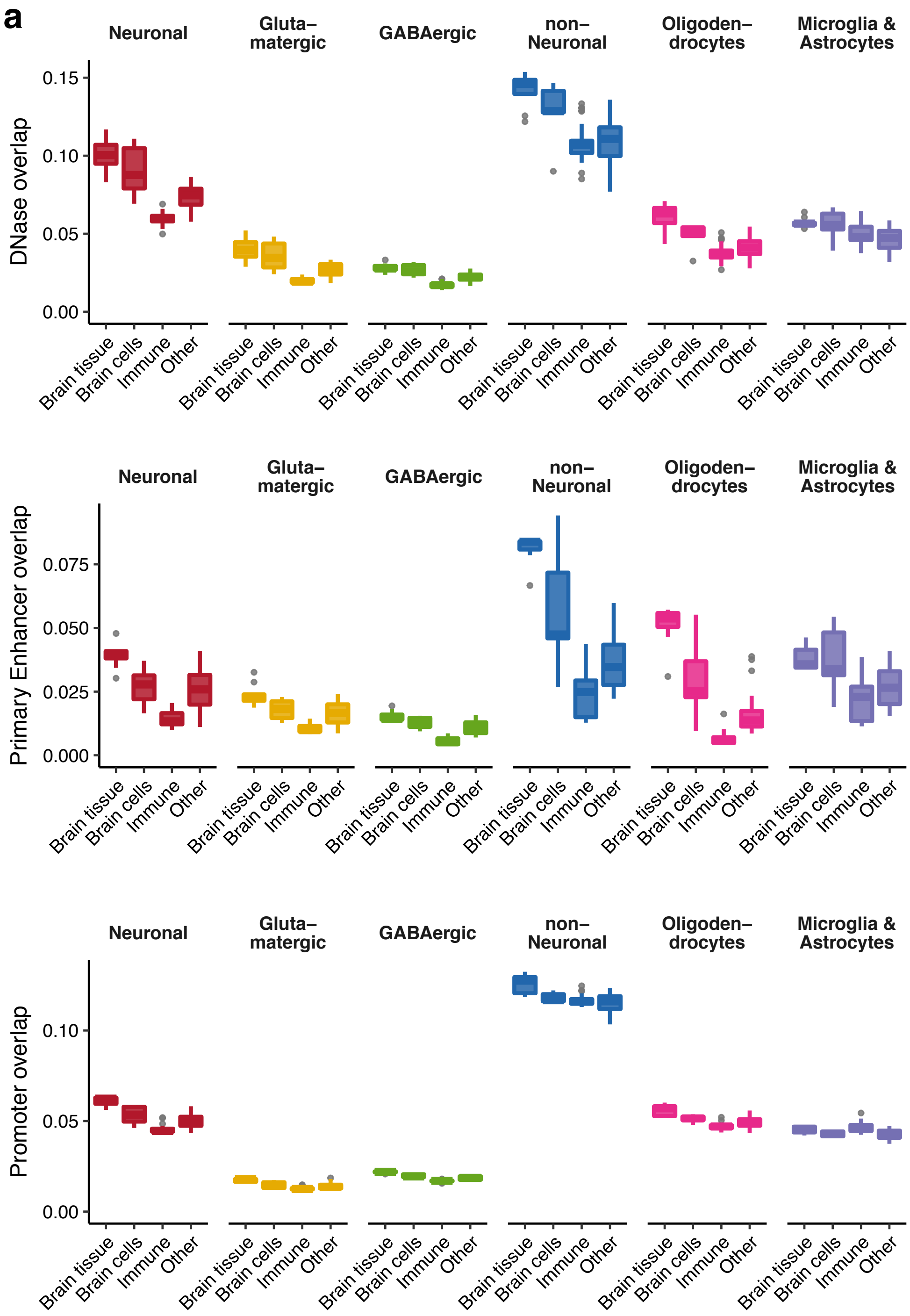
a
Neuronal
Gluta–matergic
GABAergic
non–Neuronal
Oligoden–drocytes
Microglia & Astrocytes
DNase overlap
0.15
0.10
0.05
0.00
Brain tissue
Brain cells
Immune
Other
Primary Enhancer overlap
0.075
0.050
0.025
0.000
Promoter overlap
0.10
0.05
0.00

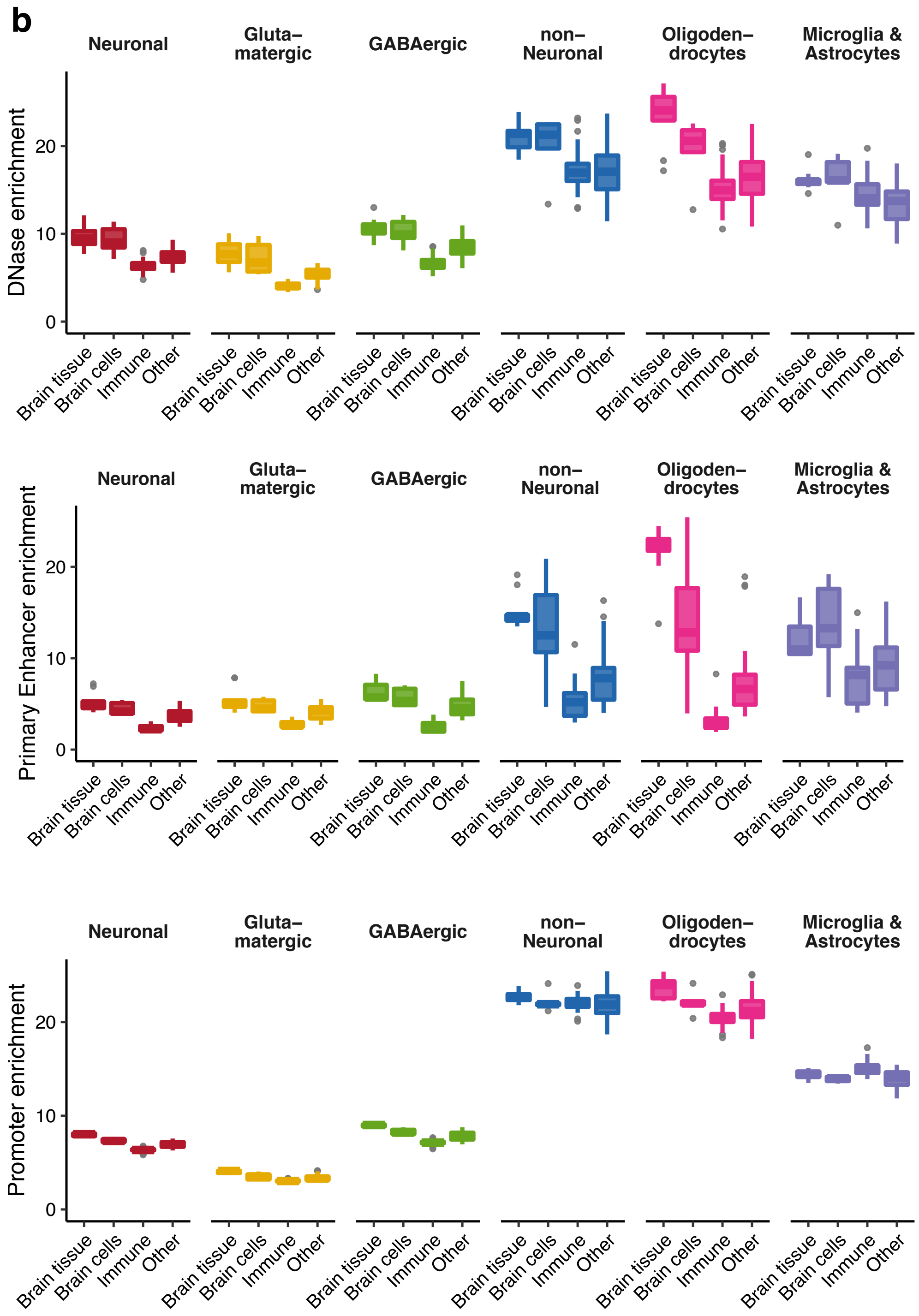

Supplementary Figure 7. Overlap of Identified cell-specific open chromatin regions with existing epigenomic annotations. The cell-specific OCRs were identified as detailed in main text. The overlap was calculated by **(a)** the Jaccard index of the base pair overlap and **(b)** enrichment against the genomic background. Samples from REMC were aggregated into 4 groups: brain tissue, brain derived cells, immune cells/tissue, and other non-brain cells/tissues. The enrichment in panel b was also recalculated after shuffling the peaks. For the 2,286 overlaps comparison jointly assessed in this manner, the median enrichment was 0.97 (min 0.69, max 1.16, interquartile range 0.95-1.00). Note that the REMC open chromatin/chromHMM states are all identified in the given cell/tissue and not those specific to it. The center shows the median, the box shows the interquartile range, whiskers indicate the highest/lowest values within 1.5x the interquartile range, and outliers from this are shown as dots. The number of independent sample overlap represented by the boxplot groups are as follows: Brain tissue: 10, Brain cells: 6, Immune: 30, and Other: 81

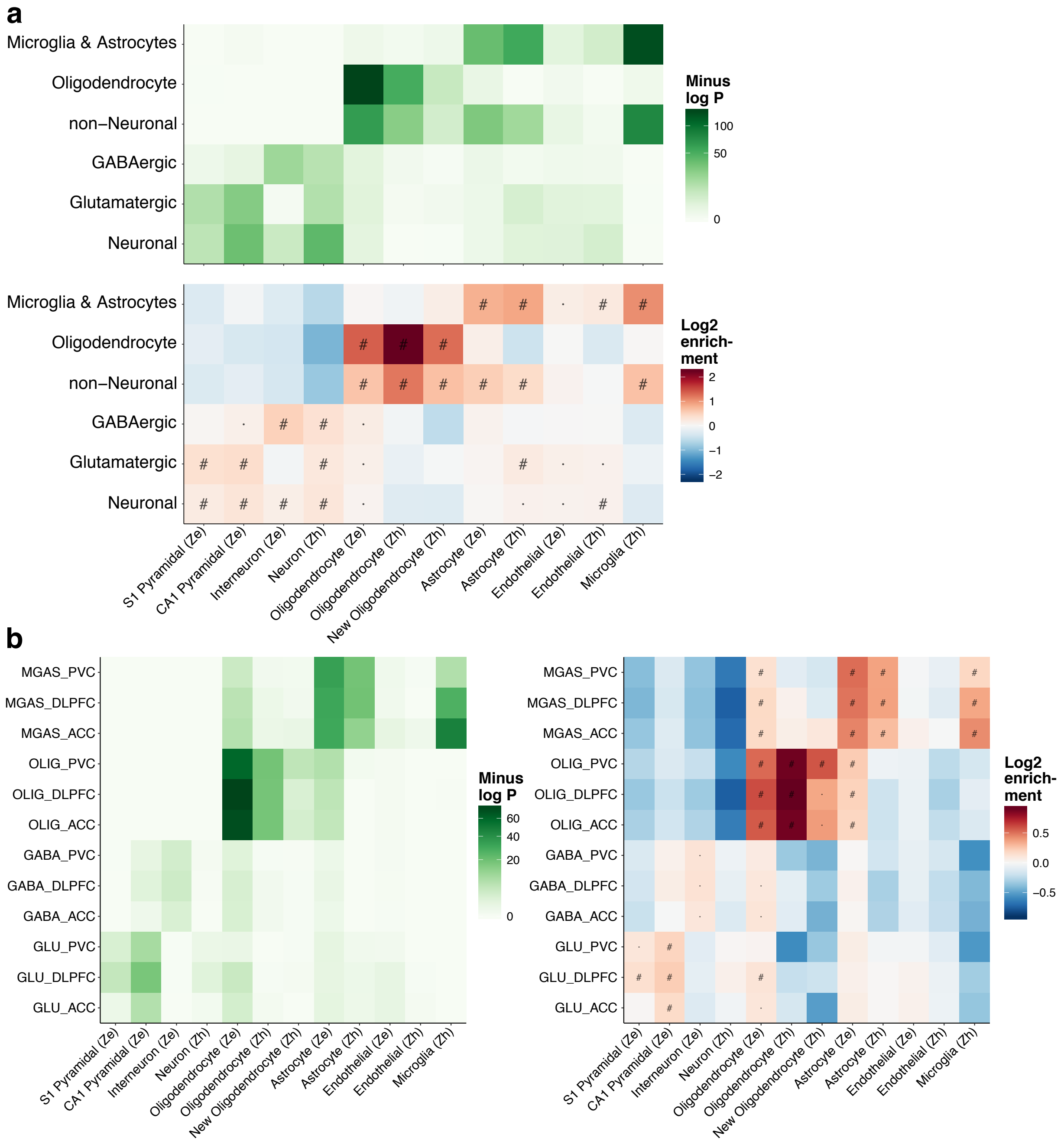

Supplementary Figure 8. Overlap between open chromatin and genes with established cell-specific expression. (a) Overlap between cell-specific OCRs (ATAC-seq) and the regulatory domains of genes with established cell-specific marker genes (RNA-seq). **(b)** Overlap between OCRs identified in all merged samples (ATAC-seq) and the regulatory domains of genes with established cell-specific marker genes (RNA-seq). "#": one-sided binomial FDR < 0.001; " · ": one-sided binomial FDR < 0.05; GLU: glutamatergic neurons; GABA: GABAergic neurons; OLIG: oligodendrocytes; MGAS: microglia and astrocytes; ACC: anterior cingulate cortex; DLPFC: dorsolateral prefrontal cortex; and PVC: primary visual cortex.

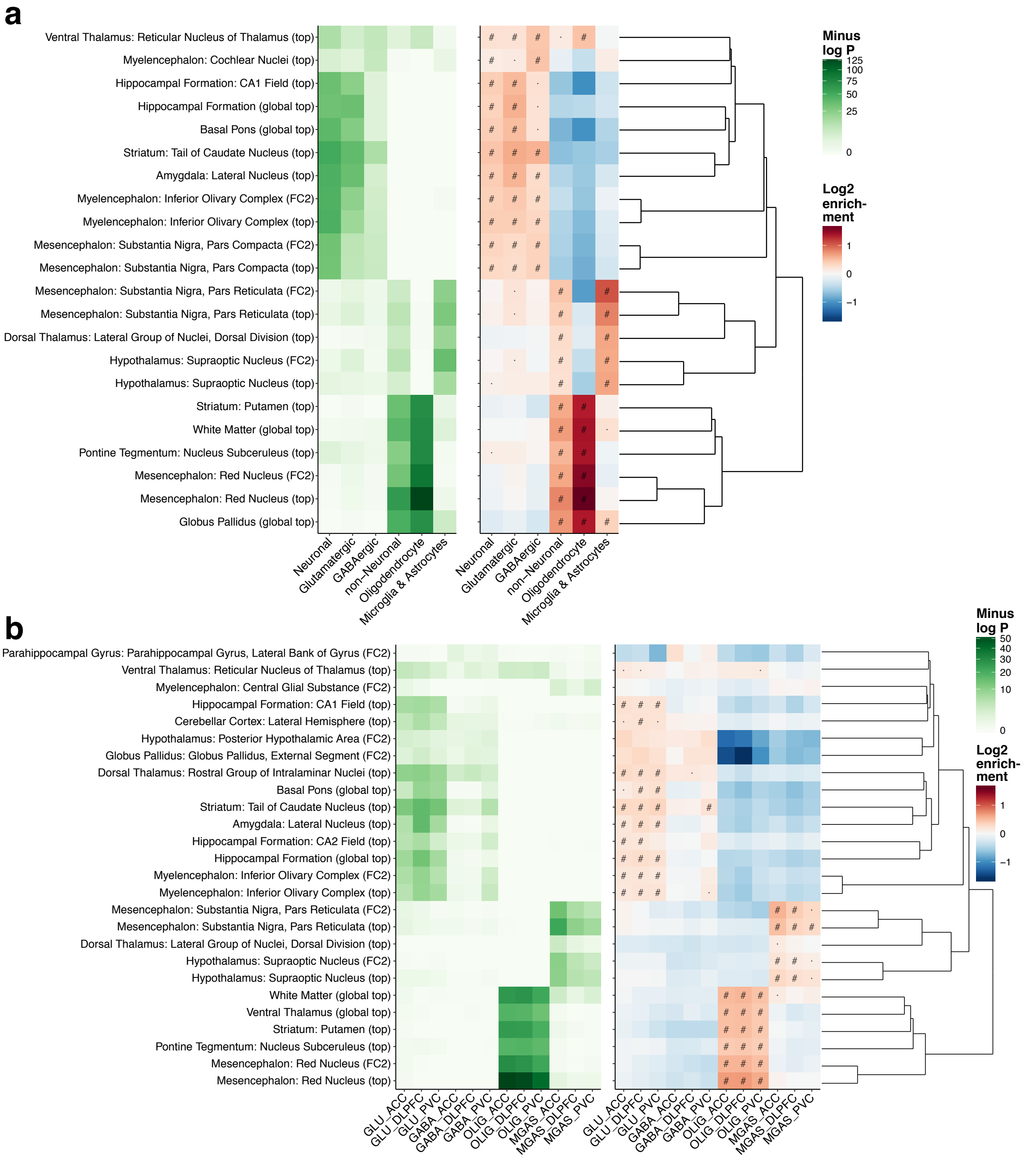

Supplementary Figure 9. Overlap between open chromatin and genes showing expression in specific brain regions. (a) Overlap between cell-specific OCRs (ATAC-seq) and the regulatory domains of genes showing increased expression in various brain regions identified by analysis of bulk tissue gene expression. **(b)** Overlap between OCRs identified in all merged samples (ATAC-seq) and the regulatory domains of genes showing increased expression in various brain regions identified by analysis of bulk tissue gene expression. . Pathways were clustered by the Jaccard index using the WardD method. "#":one-sided binomial FDR < 0.001; " · ": one-sided binomial FDR < 0.05; "top": at most genes 200 with increased expression in the brain region (Online Methods); "FC2": genes showing at least a two-fold difference in expression compared to other regions; GLU: glutamatergic neurons; GABA: GABAergic neurons; OLIG: oligodendrocytes; MGAS: microglia and astrocytes; ACC: anterior cingulate cortex; DLPFC: dorsolateral prefrontal cortex; and PVC: primary visual cortex.

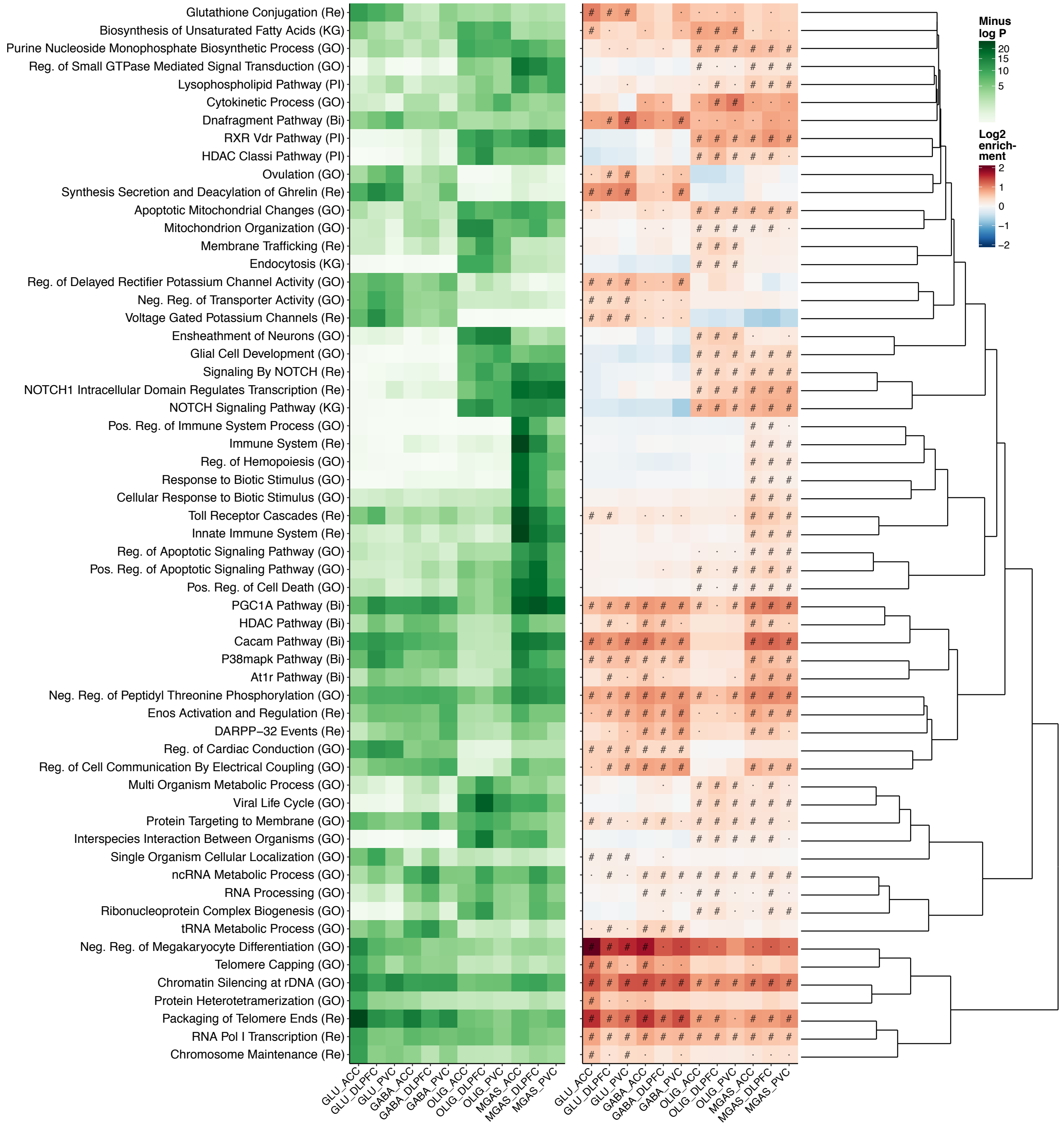

Supplementary Figure 10. Overlap between open chromatin and genes involved in specific biological functions. Overlap between OCRs identified in all merged samples (ATAC-seq) and the regulatory domains of genes showing increased expression in various biological functions. Pathways were clustered by the Jaccard index using the WardD method. “#”:one-sided binomial FDR < 0.001; “ · ”: one-sided binomial FDR < 0.05; “Bi”: BIOCARTA; “GO”: gene ontology; “KG”: KEGG; “Re”: REACTOME; GLU: glutamatergic neurons; GABA: GABAergic neurons; OLIG: oligodendrocytes; MGAS: microglia and astrocytes; ACC: anterior cingulate cortex; DLPFC: dorsolateral prefrontal cortex; and PVC: primary visual cortex, “Reg.”: regulation, “Pos.”: positive, “Neg.”: negative.

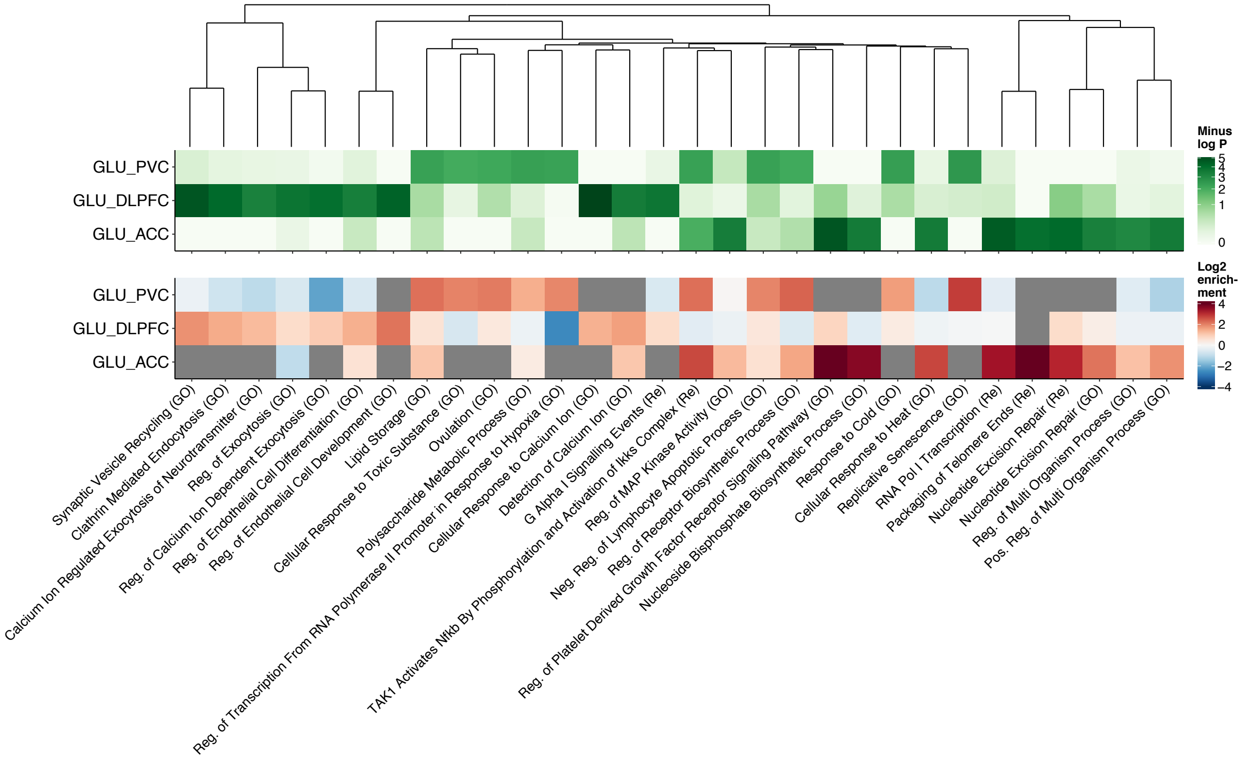

Supplementary Figure 11. Overlap between glutamatergic open chromatin with regional specificity and genes involved in specific biological functions. Overlap between brain region specific glutamatergic open chromatin (ATAC-seq) and gene sets representing biological processes and pathways. Only those that were within the top 10 most significant gene sets in one or more ATAC-seq categories are shown. Pathways were clustered by the Jaccard index using the WardD method. No gene set was significant after correcting for multiple testing. The lack of significant findings could potentially be caused by lack of power or the regionally specific glutamatergic OCRs not being properly described by current pathway annotations. “Bi”: BIOCARTA; “GO”: gene ontology; “KG”: KEGG; “Re”: REACTOME, “Reg.”: regulation, “Pos.”: positive, “Neg.”: negative

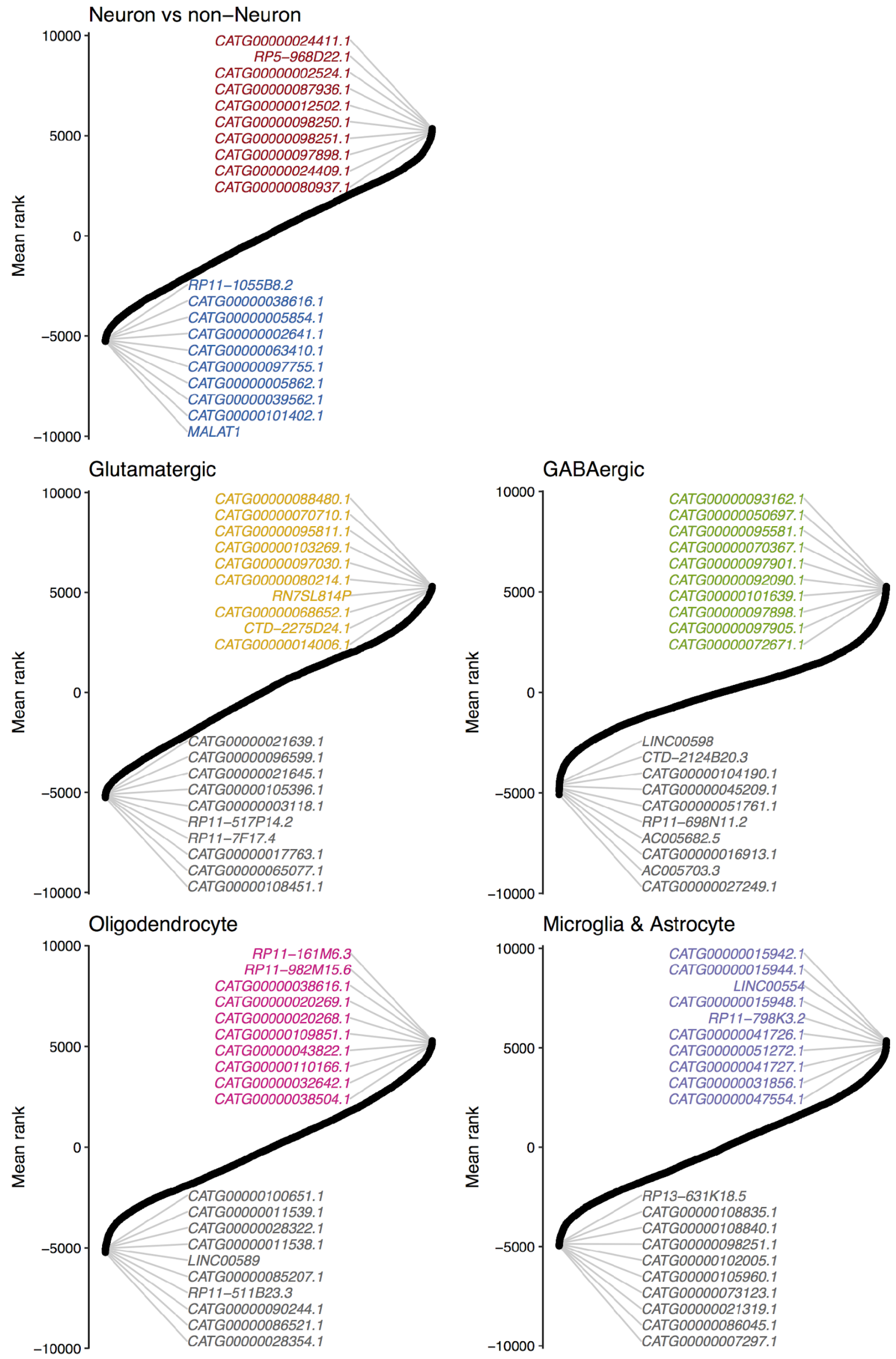

Supplementary Figure 12. Identification of cell- and region-specific regulation of lincRNA. Aggregated ranking of pairwise comparisons of lincRNA regulation between 1: neuronal/non-neuronal cells with positive values indicating a higher burden of gene regulation in neuronal samples and negative values indicating a higher burden in non-neuronal cells. 2: each of the four different cell types each compared to the remaining three cell types. Positive values indicate a higher burden of gene regulation in the given cell type and negative value a lower burden of gene regulation than in the other cell types. For the lincRNA genes we used the FANTOM CAT Robust category, from which we only considered genes from the category “far from protein coding genes” and excluded genes with coding status “uncertain”. Ultimately, 10,717 lincRNA genes were considered.

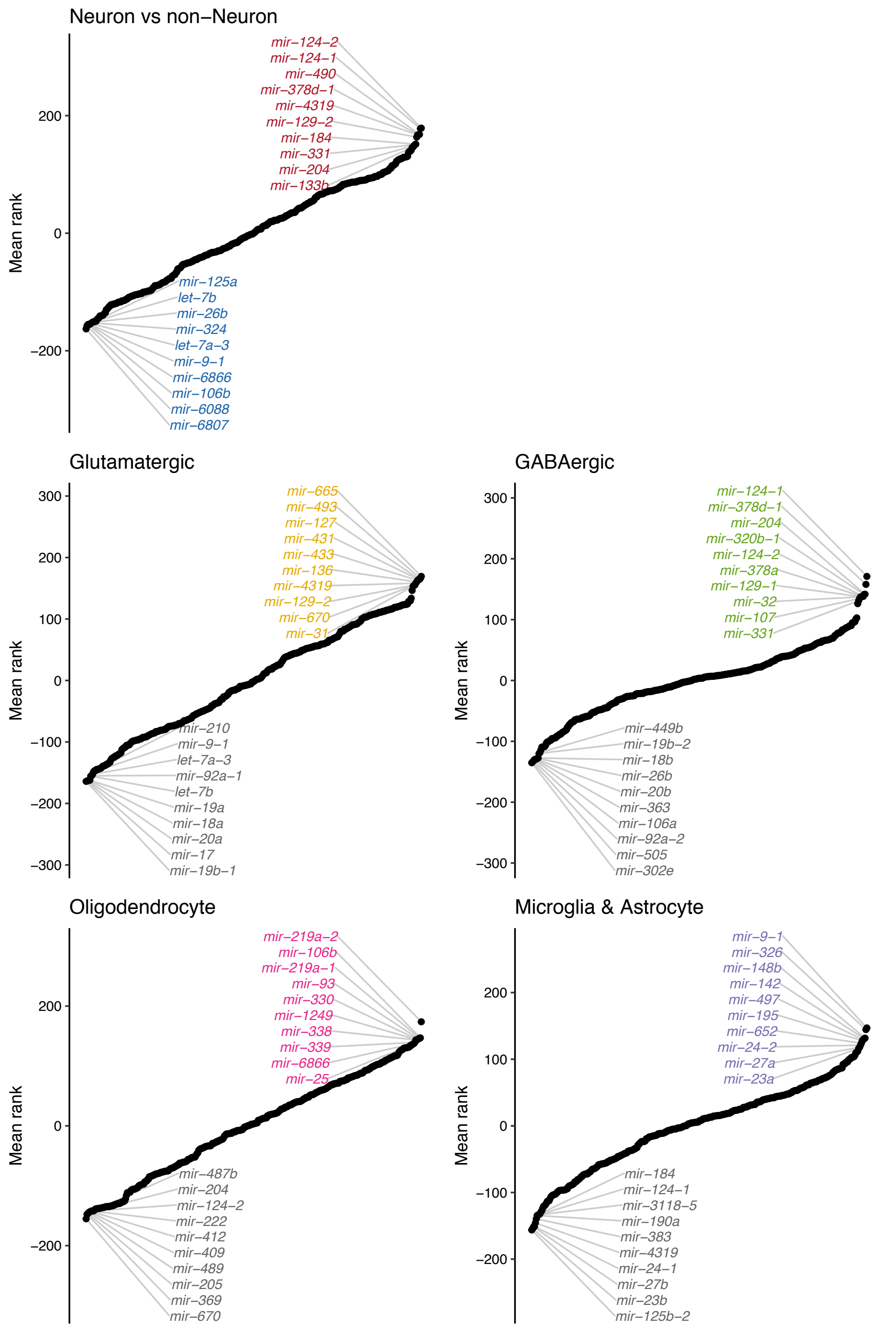

Supplementary Figure 13. Identification of cell- and region-specific regulation of microRNAs. Aggregated ranking of pairwise comparisons of microRNA regulation between 1: neuronal/non-neuronal cells with positive values indicating a higher burden of gene regulation in neuronal samples and negative values indicating a higher burden in non-neuronal cells. 2: each of the four different cell types each compared to the remaining three cell types. Positive values indicate a higher burden of gene regulation in the given cell type and negative value a lower burden of gene regulation than in the other cell types. To limit the possibility of erroneous annotations only genes encoding microRNA that were members of a conserved microRNA family were retained (n=367).

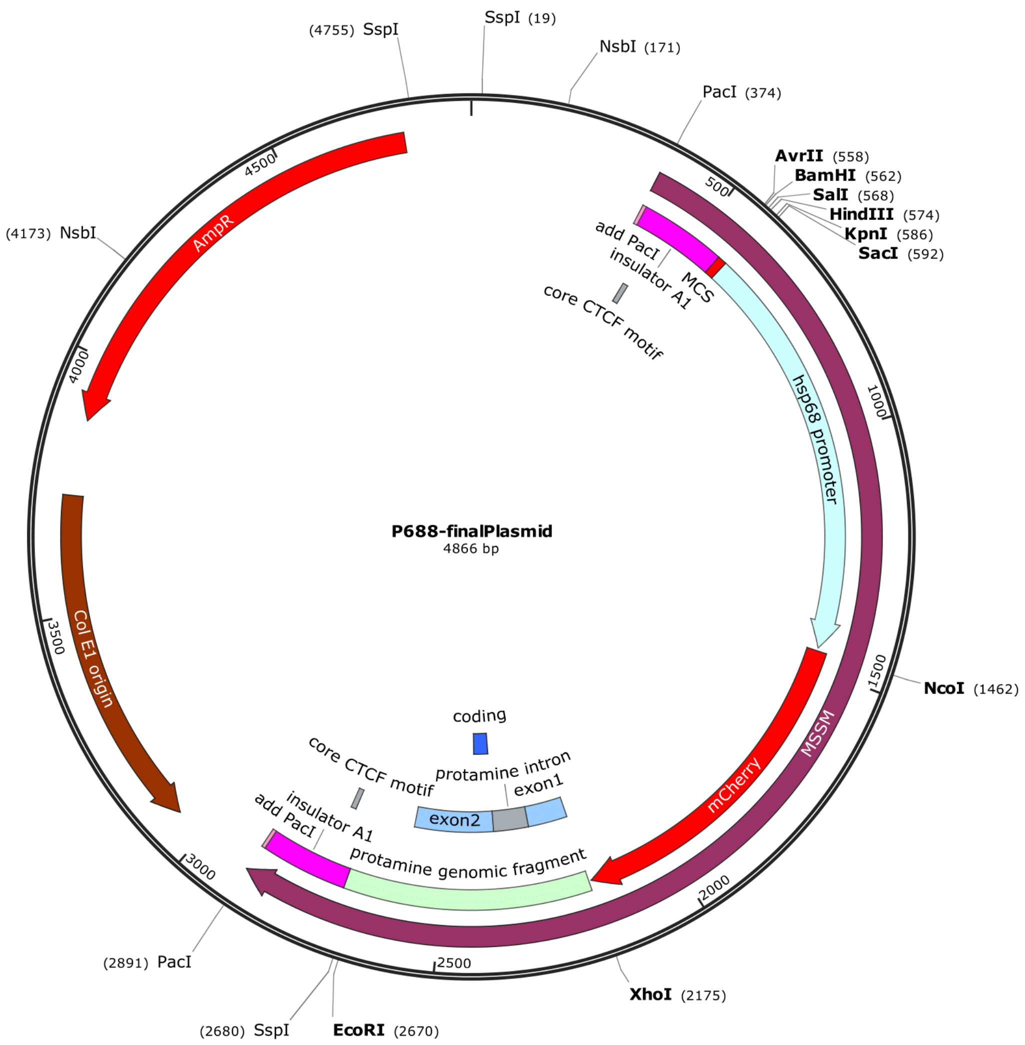

Supplementary Figure 14. Vector used for mouse transgenesis. Positions of important restriction sites are shown: sites in bold are unique cutters. MSSM: Mount Sinai School of Medicine (to delineate transgenic vector from plasmid vector); MCS: Multiple Cloning Site; ColE1: origin of replication derived from ColE1, a plasmid found in bacteria"; AmpR: ampicillin resistance.

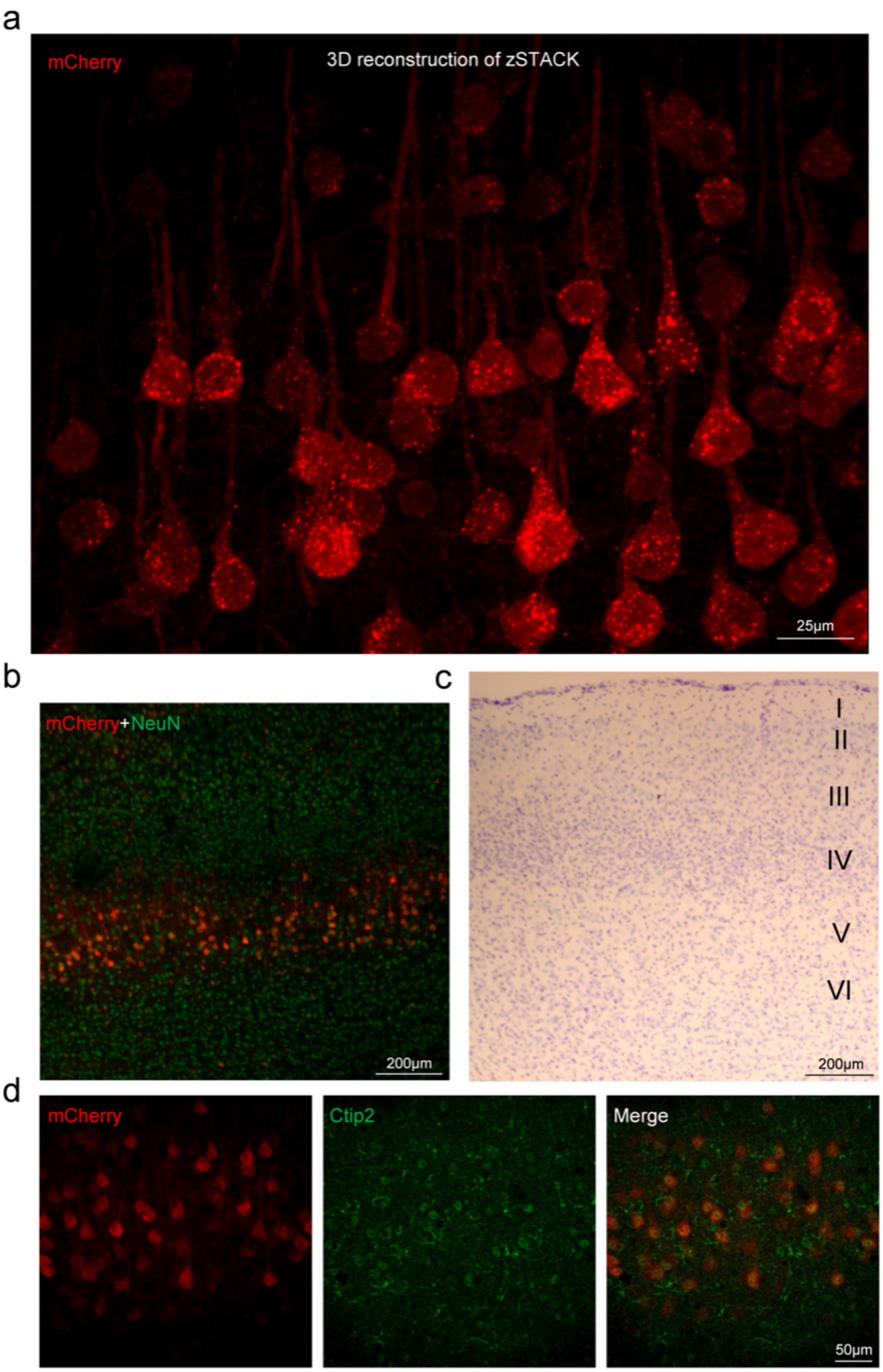

Supplementary Figure 15. In the cortex, *BNDF* OCR driven expression is restricted to layer V. (**a**) 3D zSTACK reconstruction of layer V neurons expressing mCherry from *BNDF* OCR, a putative enhancer, in transgenic mice. (**b**) Representative image showing co-localization between mCherry (red) and NeuN (green) in sagittal cortical section from *BDNF*-mCherry transgenic mice. (**c**) Representative Cresyl violet image showing the cortical layers. (**d**) Double staining representative image showing mCherry and Bcl11b/Ctip2 co-localization in layer V of the cortex in *BDNF* transgenic mice. Four image frames of three independent brain slices per each mouse were analyzed (n = 5).

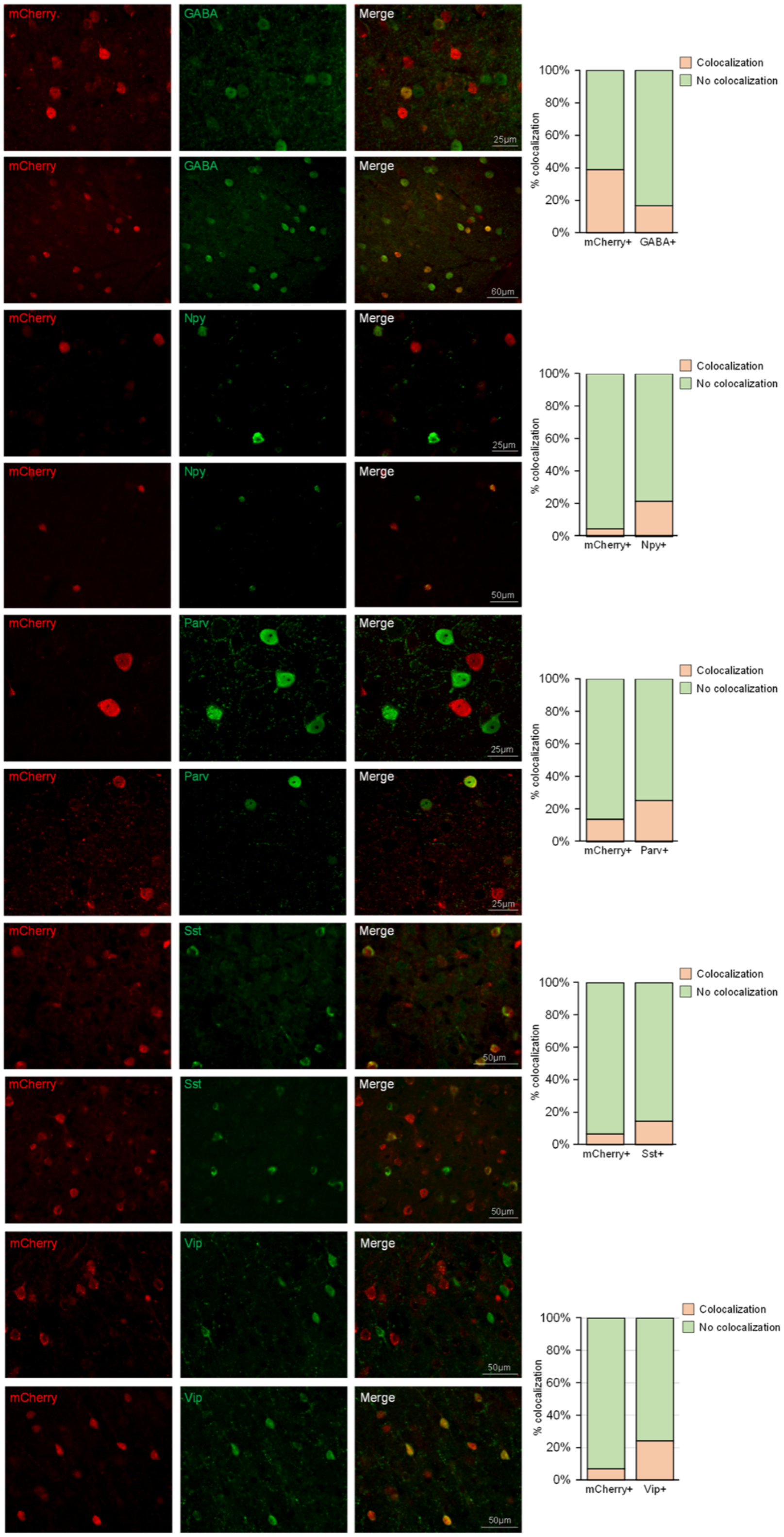

Supplementary Figure 16. mCherry expression driven by *DLX6* OCR is apparent in multiple interneuron subtypes. Representative cortical images immune-labeled with mCherry and markers of GABAergic interneuron subtypes, and graphs detailing corresponding quantification of co-labelling are shown. From top to bottom, GABA, neuropeptide Y (NPY), parvalbumin (Parv), somatostatin (Sst), and vasoactive intestinal peptide (VIP), are shown. Note that percentages vary between transgenic founders. Four image frames of three independent brain slices per each mouse were analyzed (n = 6).

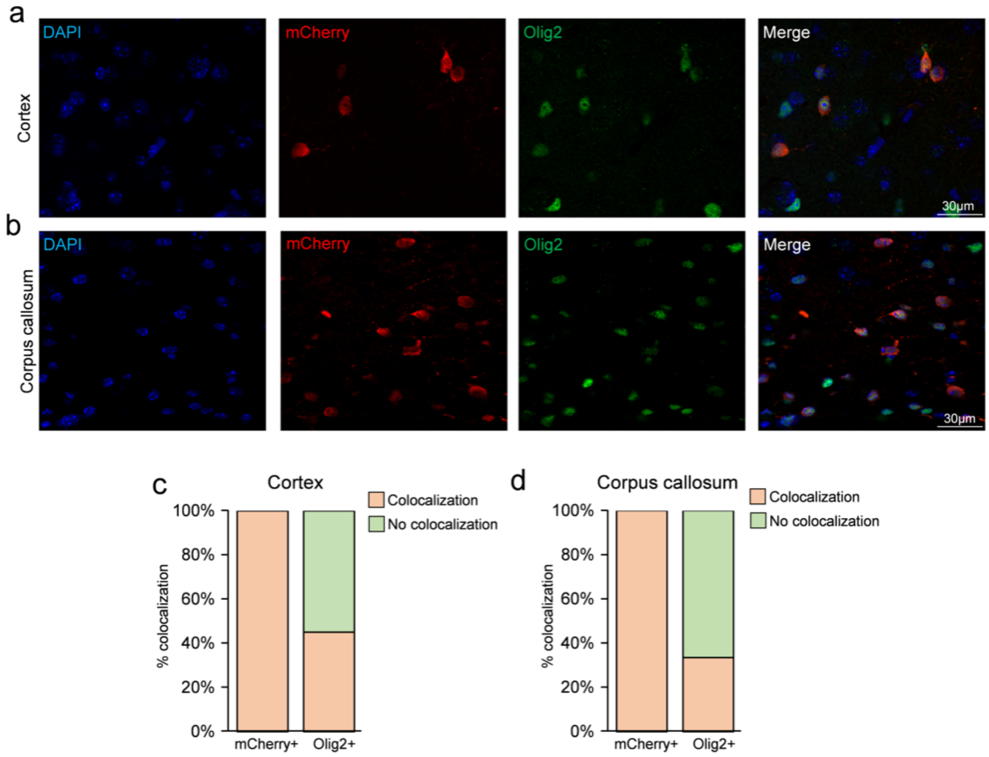

Supplementary Figure 17. The *CNDP* OCR-driven expression is restricted to oligodendrocytes. (**a-b**) Double labeling representative images from *CNDP1* transgenic mice showing co-localization between mCherry (red) and Olig2 (green) in the (**a**) cortex and (**b**) corpus callosum. (**c-d**) Graphs showing the percentage of co-localization between mCherry and Olig2 double in the (**c**) cortex and the (**d**) corpus callosum. Importantly, all mCherry-immunopositive cells also express Olig2, but mCherry is not expressed in all Olig2 cells. Four image frames of three independent brain slices per each mouse were analyzed (n = 4)

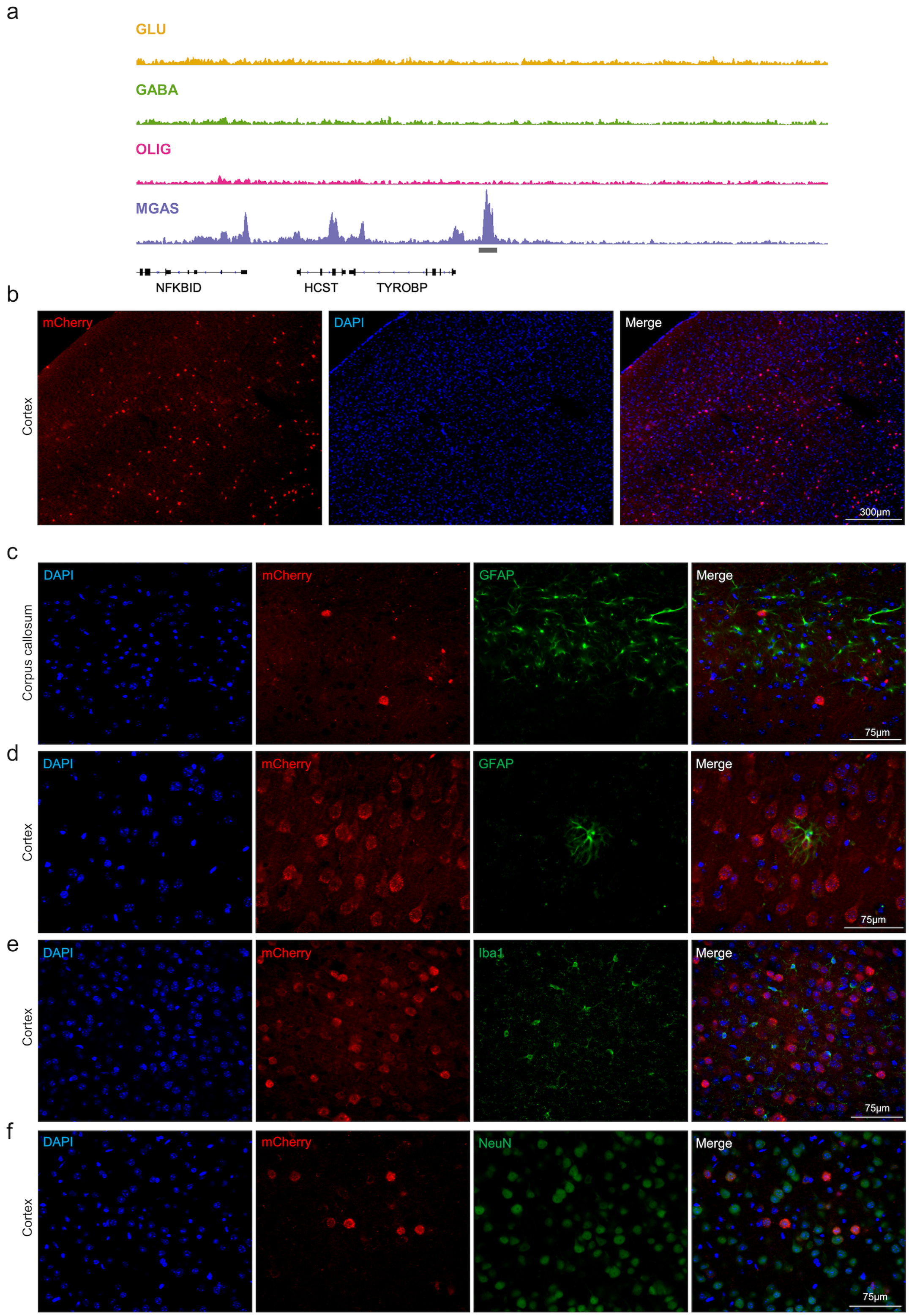

Supplementary Figure 18. *TYROBP* enhancer fails to drive expression in glial cells. (**a**) Comparison of the *TYROBP* OCR accessibility across cell types. (**b**) Representative image of mCherry (red) and DAPI (blue) double labeling in cortical region of a sagittal section from *TYROBP* transgenic mice. (**c-e**) Representative images showing lack of co-localization between mCherry and astrocytes (GFAP) and microglial cells (Iba1). (**f**) Representative image showing co-localization between mCherry (red) and NeuN (green) in the cortex of *TYROBP* transgenic mice, showing that this OCR drives expression in neurons. Four image frames of three independent brain slices per each mouse were analyzed (n = 4).

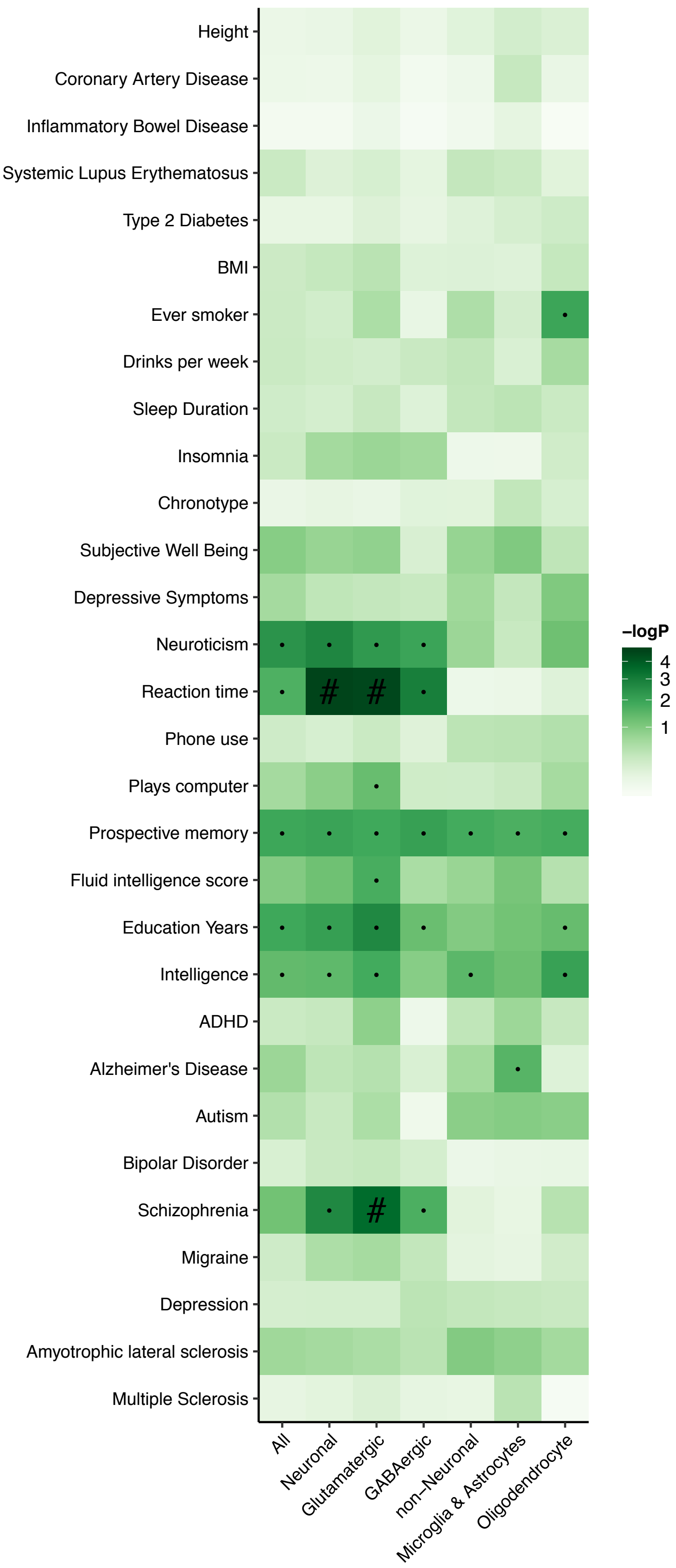

Supplementary Figure 19. Overlap between trait- and disease-associated genetic variants and all identified open chromatin regions. The overlap was assessed using LD-score partitioned heritability. Here all OCRs called in a given cell type were used to increase power. Further, the OCRs were padded with 1,000 bp to also capture adjacent genetic variants."#": Significant for enrichment in LD score regression after FDR correction of multiple testing across all traits and OCR sets (Benjamini & Hochberg); " · ": Nominally significant.

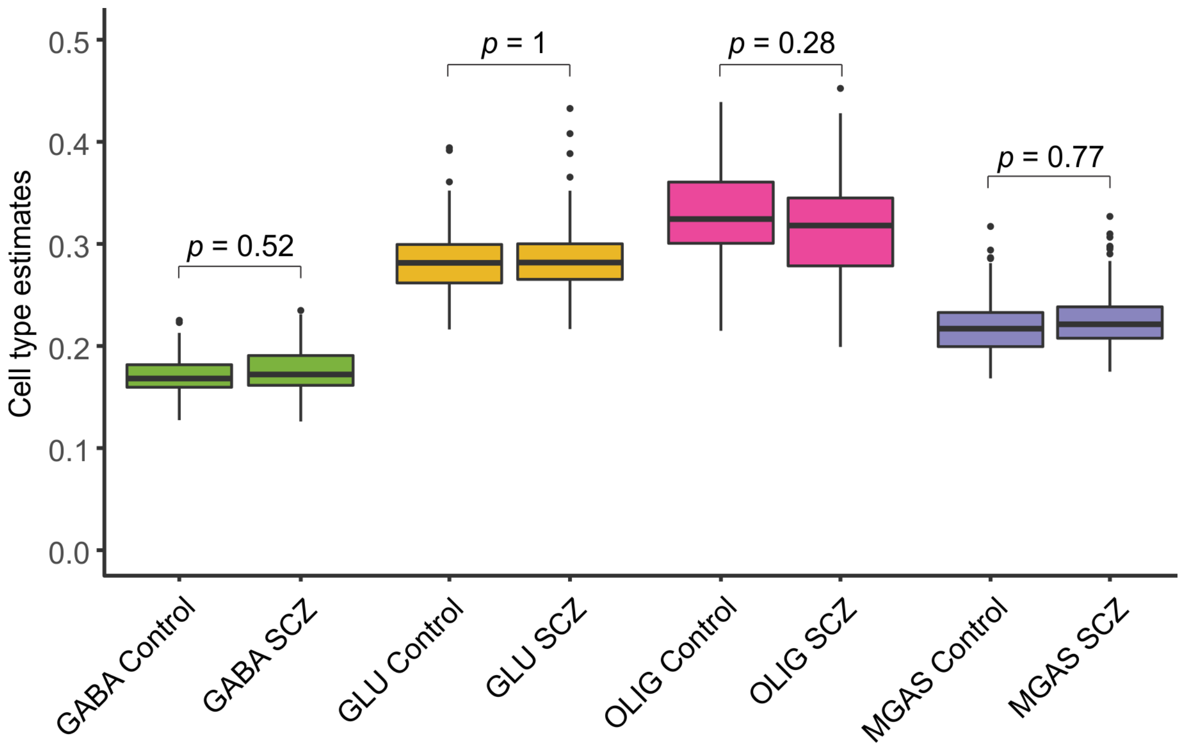

Supplementary Figure 20. Predicted cell type ratios of homogenate ATAC-seq samples from prefrontal cortex of schizophrenia cases and controls. These estimates calculated by dTangle method are based on the patterns of expression in marker OCRs derived from our cell type-specific study. The differences between schizophrenia cases and controls are not significant for any of estimated cell types as indicated by *P*-values calculated by two-sided Wilcoxon followed by adjustment for multiple testing. The center shows the median, the box shows the interquartile range, whiskers indicate the highest/lowest values within 1.5x the interquartile range, and outliers from this are shown as dots. The boxes show, respectively 126 independent controls and 121 independent cases.

Cell type and brain region	eQTL overlap with open chromatin (no padding)							eQTL overlap with padded open chromatin (%)				
	Observed				50 permutations		OR	0 bp	500 bp	1000 bp	2500 bp	5000 bp
	# of variants	# of credible	%	*P*-value	Avg.	SE						
GLU ACC	60220	1223	2.0%	1E-99	732	28	1.7	2.0%	1.6%	1.5%	1.4%	1.3%
GLU DLPFC	94261	1811	1.9%	4E-125	1160	45	1.6	1.9%	1.5%	1.4%	1.3%	1.3%
GLU PVC	62006	1339	2.2%	5E-127	742	25	1.8	2.2%	1.7%	1.5%	1.4%	1.3%
GABA ACC	44843	1211	2.7%	4E-183	519	29	2.3	2.7%	2.1%	1.9%	1.7%	1.7%
GABA DLPFC	59888	1513	2.5%	5E-201	704	33	2.2	2.5%	2.0%	1.8%	1.6%	1.6%
GABA PVC	52186	1273	2.4%	3E-158	614	29	2.1	2.4%	1.8%	1.7%	1.5%	1.4%
OLIG ACC	83115	2584	3.1%	<1E-300	948	33	2.7	3.1%	2.5%	2.3%	2.1%	2.0%
OLIG DLPFC	75980	2519	3.3%	<1E-300	865	41	2.9	3.3%	2.6%	2.4%	2.3%	2.2%
OLIG PVC	84644	2540	3.0%	<1E-300	967	40	2.6	3.0%	2.4%	2.2%	2.1%	2.0%
MGAS ACC	83856	2279	2.7%	<1E-300	966	37	2.4	2.7%	2.2%	2.0%	1.8%	1.7%
MGAS DLPFC	71808	2174	3.0%	<1E-300	824	34	2.6	3.0%	2.4%	2.2%	2.0%	1.9%
MGAS PVC	68625	2001	2.9%	<1E-300	786	31	2.6	2.9%	2.3%	2.0%	1.9%	1.8%

Supplementary Table 1. Enrichment of open chromatin region in eQTLs. "# of variants" indicates how many variants overlapped the annotation. "# of credible" indicates how many credible SNPs overlapped the annotation. "%" indicates the percentage over all SNPs overlapping the annotation that were credible SNPs. *P*-value is for enrichment against the background (Where 1% of SNPs were credible) using a Fisher exact test. 50 permutations were done to evaluate potential biases. Additionally, the table shows the percentages of credible SNPs where each OCR of the peak was padded to varying degrees. OCR: open chromatin region. GLU: glutamatergic neurons; GABA: GABAergic neurons; OLIG: oligodendrocytes; MGAS: microglia and astrocytes; ACC: anterior cingulate cortex; DLPFC: dorsolateral prefrontal cortex; and PVC: primary visual cortex
