# Supplementary material for: Common schizophrenia risk variants are enriched in open chromatin regions of human glutamatergic neurons
Source: Nat Commun. 2020 Nov 4;11:5581. doi: 10.1038/s41467-020-19319-2 (PMC7643171; doi:10.1038/s41467-020-19319-2)
Supplement: Supplementary file 3 — Description of Additional Supplementary Files [file 41467_2020_19319_MOESM3_ESM.pdf]

Description of Additional Supplementary Files

Supplementary Data 1 Sample and subject level information

Supplementary Data 2 Cell-specific open chromatin regions

Supplementary Data 3 Region specific open chromatin regions in glutamatergic neurons

Supplementary Data 4 Protein coding genes, lncRNA, and microRNA with TSSs overlapping cell-specific open chromatin regions

Supplementary Data 5 Cell-specific peaks used for deconvolution of bulk ATAC-seq samples
