# Supplementary material for: Common schizophrenia risk variants are enriched in open chromatin regions of human glutamatergic neurons
Source: Nat Commun. 2020 Nov 4;11:5581. doi: 10.1038/s41467-020-19319-2 (PMC7643171; doi:10.1038/s41467-020-19319-2)
Supplement: Supplementary file 9 — Reporting summary [file 41467_2020_19319_MOESM9_ESM.pdf]

nature research

Corresponding author(s): Panos Roussos

Last updated by author(s): Aug 22, 2020

Reporting Summary

Nature Research wishes to improve the reproducibility of the work that we publish. This form provides structure for consistency and transparency in reporting. For further information on Nature Research policies, see our Editorial Policies and the Editorial Policy Checklist.

Statistics

For all statistical analyses, confirm that the following items are present in the figure legend, table legend, main text, or Methods section.

n/a	Confirmed	
☐	☒	The exact sample size (n) for each experimental group/condition, given as a discrete number and unit of measurement
☐	☒	A statement on whether measurements were taken from distinct samples or whether the same sample was measured repeatedly
☐	☒	The statistical test(s) used AND whether they are one- or two-sided<br>*Only common tests should be described solely by name; describe more complex techniques in the Methods section.*
☐	☒	A description of all covariates tested
☐	☒	A description of any assumptions or corrections, such as tests of normality and adjustment for multiple comparisons
☐	☒	A full description of the statistical parameters including central tendency (e.g. means) or other basic estimates (e.g. regression coefficient) AND variation (e.g. standard deviation) or associated estimates of uncertainty (e.g. confidence intervals)
☐	☒	For null hypothesis testing, the test statistic (e.g. F, t, r) with confidence intervals, effect sizes, degrees of freedom and P value noted<br>*Give P values as exact values whenever suitable.*
☒	☐	For Bayesian analysis, information on the choice of priors and Markov chain Monte Carlo settings
☒	☐	For hierarchical and complex designs, identification of the appropriate level for tests and full reporting of outcomes
☐	☒	Estimates of effect sizes (e.g. Cohen's d, Pearson's r), indicating how they were calculated

Our web collection on statistics for biologists contains articles on many of the points above.

Software and code

Policy information about availability of computer code

Data collection	BD FACSdiva 8.0.1, ImageJ (version 1.51)
Data analysis	ataqv 1.2.0, bamCorrelate from deepTools 3.2.1, bamUtil 1.0.14, bcftools v1.9, bedGraphToBigWig v4.0.0, BEDTools v2.25.0, ChIPSeeker 1.2.6, edgeR 3.14.0, fastqc v0.11.2, gatk v3.5-0, GenomicFeatures 1.24.2, limma 3.28.4, java v1.7.0_60, KING 1.9, LDSc 1.0.0, MACS v2.1, phantompeakqualtools v2.0, picard v2.2.4, PIQ 1.3, plink v1.90, python v2.7.14, qualimap v2.0, R v3.5.3, Rtsne 0.13, samtools v1.3, star v2.5.0, vcftools v0.1.14-10, wiggletools 1.0, and wigToBigWig v4.0.0

For manuscripts utilizing custom algorithms or software that are central to the research but not yet described in published literature, software must be made available to editors and reviewers. We strongly encourage code deposition in a community repository (e.g. GitHub). See the Nature Research guidelines for submitting code & software for further information.

Data

Policy information about availability of data

All manuscripts must include a data availability statement. This statement should provide the following information, where applicable:

- Accession codes, unique identifiers, or web links for publicly available datasets
- A list of figures that have associated raw data
- A description of any restrictions on data availability

The ATAC-seq data generated as part of this publication have been deposited in Gene Expression Omnibus and are accessible through GEO Series accession number GSE143666. Further, UCSC tracks and downloads are provided at our webpage: http://icahn.mssm.edu/boca2

Further, the following online resources and databases were used: dbSNP: https://www.ncbi.nlm.nih.gov/snp, REMC: http://www.roadmapepigenomics.org, MSigDB (GO, KEGG, and Biocarta gene sets): https://www.gsea-msigdb.org, lincRNA and microRNA from FANTOM: https://fantom.gsc.riken.jp, and miRBase: http://

www.mirbase.org.

Field-specific reporting

Please select the one below that is the best fit for your research. If you are not sure, read the appropriate sections before making your selection.

- [x] Life sciences
- [ ] Behavioural & social sciences
- [ ] Ecological, evolutionary & environmental sciences

For a reference copy of the document with all sections, see nature.com/documents/nr-reporting-summary-flat.pdf

Life sciences study design

All studies must disclose on these points even when the disclosure is negative.

Sample size

Human brain samples: 48. Mice:33, which was considered relevant based on previous experiments (PMID: 29945882)

Data exclusions

During quality control, one ACC derived GABA ATAC-seq sample was found to be of poor quality as it showed a low final read count, a low fraction of reads in peaks and was an outlier in a clustering analysis. This sample was excluded and the remaining samples were reprocessed through the pipeline. The sample is thus excluded from all analyses.

Replication

Humans: multiple samples from the same brain-region and cell type were assayed in parrallel. In particular four human brains were assayed in three different brain regions, from where nuclei from four distinct cell type were isolated. Data analyses including clustering and visual inspection showed the results to be replicable.

Mice: Multiple founders for each transgene were assayed (numbers detailed in Table 2). A minimum of three brain slices from different brain regions per each mouse were assayed. In addition, results were compared to existing data sources when available. mCherry expression and cell-type specificity inspection showed the results to be replicable. Number of mice with reporter expression: BDNF enhancer 5 of 6; DLX6 enhancer 6 of 11; CNDP1 enhancer 4 of 6; TYROBP enhancer 4 of 5.

Randomization

Generation of ATAC-seq libraries were randomized for sample ID, brain region and cell type at multiple steps including:
1. During FANS and transposase reaction
2. PCR amplification for library preparation/barcode assignment
3. Gel extraction
4. Sequencing pools

Blinding

Human samples: Not relevant, as this was not a case control design. All samples were a priori believed to yield similar results.

Mice: For each pronuclear injection, only ~25% of mice incorporated the vector. We included only a single non-transgenic mice in our immunofluorescence studies to demonstrate specificity of staining. For this reason, the experiments were not blinded.

Reporting for specific materials, systems and methods

We require information from authors about some types of materials, experimental systems and methods used in many studies. Here, indicate whether each material, system or method listed is relevant to your study. If you are not sure if a list item applies to your research, read the appropriate section before selecting a response.

Materials & experimental systems

n/a	Involved in the study	
[ ]	[x]	Antibodies
[x]	[ ]	Eukaryotic cell lines
[x]	[ ]	Palaeontology and archaeology
[ ]	[x]	Animals and other organisms
[ ]	[x]	Human research participants
[x]	[ ]	Clinical data
[x]	[ ]	Dual use research of concern

Methods

n/a	Involved in the study	
[x]	[ ]	ChIP-seq
[ ]	[x]	Flow cytometry
[x]	[ ]	MRI-based neuroimaging

Antibodies

Antibodies used

SORTING
NeuN antibody (1:1000, PE conjugated,Millipore; FCMAB317PE), anti-SOX6 (PMID: 26612861) and anti-SOX10 (PMID: 24561062).

HISTOLOGY
rat anti-mCherry (1:10000; Invitrogen; M11217), rabbit anti-NeuN (1:1000; Millipore; ABN78), rabbit anti-GABA (1:2000; Sigma; A2052), rabbit anti-PARV (1:1000; Swant; PV27), rabbit anti-Somatostatin (1:500; Peninsula; T-4103), rabbit anti-NPY (1:500; Abcam; ab30914), rabbit anti-VIP (1:1000; Immunostar; 20077), rabbit anti-Iba1 (1:500; WAKO; 019-19741), rabbit anti-Olig2 (1:500; Abcam;

	ab136253), rabbit anti-Ctip2 (aka Bcl11b; 1:500; Bethyl Laboratories; A300-385A), and rabbit anti-GFAP (1:500; DAKO; Z0334)

Validation

SORTING
NeuN antibody: PE-conjugated mouse anti-NeuN (1:1000; Millipore; FCMAB317PE). This particular clone (A60) has been referenced in 32 publications, according to the manufacturer's website, and has been extensively used in the isolation of neuronal nuclei(PMID: 18442397, PMID: 29945882).

SOX6 antibody: Goat anti-sox 10 (1:300; R&D Systems, AF2864). Has been used for the isolation of oligodendrocyte nuclei in several publications(PMID: 30263963, PMID: 26612861)

SOX10 antibody: Guinea-pig anti-sox 6 (1:1500) has been used to isolate GABAergic neuronal nuclei in a number of publications (PMID: 24561062, PMID: 30263963, PMID: 22632721).

HISTOLOGY
mCherry antibody: Antibody has been referenced in 61 publications according to the manufacturer's website. Including Wang et al., 2019; eLife (doi.org/10.7554/eLife.43999)

NeuN antibody: Antibody has been referenced in several publications according to the manufacturer's website. Including Lundgaard et al., 2015; Nature Communications (doi.org/10.1038/ncomms7807)

GABA antibody: Antibody has been referenced in 411 publications according to the manufacturer's website. Including Adhikari et al., 2015; Nature (doi.org/10.1038/nature15698)
Parvalbumin antibody: Antibody has been validated by the manufacturer and has been referenced in 10 publications. Including Owen et al., 2018; Cell (doi.org/10.1016/j.cell.2018.01.005)

Somatostatin antibody: Antibody has been validated by the manufacturer and referenced in multiple publications. Including Xu et al., 2013; Neuron (doi.org/10.1016/j.neuron.2012.11.004)

Neuropeptide Y antibody: Antibody has been referenced in 42 publications according to the manufacturer's website. Including Cheng et al., 2019; Cell Reports (doi.org/10.1016/j.celrep.2019.02.068)

VIP antibody: Antibody has been referenced in multiple publications according to the manufacturer's website. Including Patton et al., 2020; Nature Communications (doi.org/10.1038/s41467-020-17110-x)

Iba1 antibody: Antibody has been referenced in multiple publications according to the manufacturer's website. Including Lalancette-Hébert et al., 2012; Journal of Neuroscience (doi.org/10.1523/jneurosci.1498-12.2012)

Olig2 antibody: Antibody has been validated by the manufacturer and referenced in 2 publications. It behaved as expected by marking the appropriate regions and cells consistent with other publications.

Ctip2 antibody: Antibody has been referenced in 9 publications according to the manufacturer's website. It behaved as expected by marking the appropriate regions and cells consistent with other publications.

GFAP antibody: Antibody has been referenced in over 250 publications according to the manufacturer's website. Including Neumann et al., 2020; Scientific Reports (doi.org/10.1038/s41598-020-69266-7)

Animals and other organisms

Policy information about studies involving animals; ARRIVE guidelines recommended for reporting animal research

Laboratory animals	6-week-old male and female B6D2 F1 hybrid mice were used as the basis for transgenic strains. Mice were maintained at 22 +/- 2°C and 40-60% humidity with 12 h light/dark cycle, with ad libitum access to food and water
Wild animals	The study did not involve such samples
Field-collected samples	The study did not involve such samples
Ethics oversight	Experimental procedures were carried out in compliance with the United States Public Health Service's Policy on Humane Care and Use of Experimental Animals and were approved by the Institutional Animal Care and Use Committee at Icahn School of Medicine at Mount Sinai. We used an approved protocol with the number16-0847. The IACUC is LA09-00272.

Note that full information on the approval of the study protocol must also be provided in the manuscript.

Human research participants

Policy information about studies involving human research participants

Population characteristics	Four post-mortem caucasian donors, consisting of three males and one female, ages 20-28. The pathologist performing autopsy determined the cause of death. All were determined to have died a sudden, natural death. The subjects had no history of illicit substance abuse, alcohol abuse or psychiatric disorders. The subjects had a negative toxicology and were not taking neuropsychiatric medications (including benzodiazepines, anticonvulsants, any antipsychotics, antidepressants, or

	lithium).
Recruitment	The samples were collected with a post-mortem interval of less than 24 hours at autopsy from The Department of Forensic and Insurance Medicine, Semmelweis University, Hungary, as previously described. To the best of our knowledge the exclusion criteria listed above including the requirement of sudden natural death should preclude selection biases that would affect the post mortem brain analyses.
Ethics oversight	The Department of Forensic and Insurance Medicine, Semmelweis University

Note that full information on the approval of the study protocol must also be provided in the manuscript.

Flow Cytometry

Plots

Confirm that:

- [x] The axis labels state the marker and fluorochrome used (e.g. CD4-FITC).
- [x] The axis scales are clearly visible. Include numbers along axes only for bottom left plot of group (a 'group' is an analysis of identical markers).
- [x] All plots are contour plots with outliers or pseudocolor plots.
- [x] A numerical value for number of cells or percentage (with statistics) is provided.

Methodology

Sample preparation	From each dissection, 50mg of frozen brain tissue was homogenized in cold lysis buffer (0.32M Sucrose, 5 mM CaCl2, 3 mM Magnesium acetate, 0.1 mM, EDTA, 10mM Tris-HCl, pH8, 1 mM DTT, 0.1% Triton X-100) and filtered through a 40µm cell strainer. The flow-through was underlaid with sucrose solution (1.8 M Sucrose, 3 mM Magnesium acetate, 1 mM DTT, 10 mM Tris-HCl, pH8) and subjected to ultracentrifugation at 24,000 rpm for 1 hour at 4°C. Pellets were resuspended in 500µl DPBS and incubated in BSA (final concentration 0.1%) and anti-NeuN antibody (1:1000, Alexa488 conjugated, Millipore Cat #MAB377X), anti-SOX67 and anti-SOX108 as previously described4 Prior to FANS sorting, DAPI (Thermoscientific) was added to a final concentration of 1µg/ml. GABAergic neurons (DAPI+ NeuN+ SOX6+), Glutamatergic neurons (DAPI+ NeuN+ SOX6-), oligodendrocytes (DAPI+ NeuN- SOX10+) and microglia/astrocytes (DAPI+ NeuN- SOX10-) nuclei were sorted into individual tubes (pre-coated with 5% BSA) using a FACSAria flow cytometer (BD Biosciences).
Instrument	FACSAria flow cytometer (BD Biosciences).
Software	BD FACSDiva software (BD Biosciences)
Cell population abundance	GABAergic 5.68%<br>Glutamatergic 31.5%<br>MicrogliaAndAstrocytes 10.8%<br>Oligodendrocyte 52.0%<br><br>Samples were subsequently counted to confirm yields using a Countess II cell counter (Life Technologies)
Gating strategy	Nuclear populations were initially gated by side and forward scatter to differentiate nuclei from cellular debris.<br>Populations were then gated based on DAPI staining to identify singlets and to further remove debris.<br>DAPI positive nuclei were subsequently gated based on NeuN staining to differentiate neurons from non-neurons and, finally, these two subpopulations were further gated based on SOX6 and SOX 10 staining to identify GABAergic neurons (DAPI+ NeuN + SOX6+), Glutamatergic neurons (DAPI+ NeuN+ SOX6-), oligodendrocytes (DAPI+ NeuN- SOX10+) and microglia/astrocytes (DAPI+ NeuN- SOX10-) nuclei.

- [x] Tick this box to confirm that a figure exemplifying the gating strategy is provided in the Supplementary Information.
